# Impact of Sex in the Efficacy of Perioperative Desensitization Procedures in Heart Transplantation: A Retrospective Cohort Study

**DOI:** 10.3389/fimmu.2021.659303

**Published:** 2021-07-07

**Authors:** Lee S. Nguyen, Joe-Elie Salem, Marie-Cécile Bories, Guillaume Coutance, Julien Amour, Adrien Bougle, Caroline Suberbielle, Vissal-David Kheav, Maryvonnick Carmagnat, Philippe Rouvier, Matthias Kirsch, Shaida Varnous, Pascal Leprince, Samir Saheb

**Affiliations:** ^1^ Sorbonne Université, Department of Cardiothoracic Surgery, AP.HP.6 Pitie-Salpetriere, Paris, France; ^2^ CMC Ambroise Paré, Research and Innovation, RICAP, Neuilly-sur-Seine, France; ^3^ Sorbonne Université, Clinical Investigations Center, AP.HP.6, INSERM, Paris, France; ^4^ Jacques Cartier Private Hospital, Department of Cardiothoracic Surgery, Massy, France; ^5^ Sorbonne Université, Department of Anesthesiology, AP.HP.6 Pitie-Salpetriere, Paris, France; ^6^ Saint-Louis Hospital, Immunology Department, Paris, France; ^7^ Sorbonne Université, Department of Anatomopathology, AP.HP.6 Pitie-Salpetriere, Paris, France; ^8^ Department of Cardiovascular Surgery, University Hospital, Lausanne, Switzerland; ^9^ Sorbonne Université, service d’hémobiologie, AP.HP.6 Pitie-Salpetriere, Paris, France

**Keywords:** heart transplant, antibody mediated allograft rejection, desensitization, sex influence, gender inequalities

## Abstract

**Background:**

Sensitized patients, i.e. recipients with preformed donor-specific HLA antibodies (pfDSA), are at high-risk of developing antibody-mediated rejections (AMR) and dying after heart transplantation (HTx). Perioperative desensitization procedures are associated with better outcomes but can cause sensitization, which may influence their efficacy.

**Methods:**

In sensitized patients (pfDSA>1000 mean immunofluorescence (MFI) units), we assessed the effect of perioperative desensitization by comparing treated patients to a historical control cohort. Multivariable survival analyses were performed on the time to main outcome, a composite of death and biopsy-proven AMR with 5-year follow-up.

**Results:**

The study included 68 patients: 31 control and 37 treated patients. There was no difference in preoperative variables between the two groups, including cumulative pfDSA [4026 (1788;8725) *vs* 4560 (3162;13392) MFI units, *p*=0.28]. The cause of sensitization was pregnancy in 24/68, 35.3%, transfusion in 61/68, 89.7%, and previous HTx in 4/68, 5.9% patients. Multivariable analysis yielded significant protective association between desensitization and events (adjusted (adj.) hazard ratio (HR)=0.44 (95% confidence interval (95CI)=0.25-0.79), *p*=0.006) and deleterious association between cumulative pfDSA and events [per 1000-MFI increase, adj.HR=1.028 (1.002-1.053), p=0.031]. There was a sex-difference in the efficacy of desensitization: in men (n=35), the benefit was significant [unadj.HR=0.33 (95CI=0.14-0.78); p=0.01], but not in women (n=33) [unadj.HR=0.52 (0.23-1.17), p=0.11]. In terms of the number of patients treated, in men, 2.1 of patients that were treated prevented 1 event, while in women, 3.1 required treatment to prevent 1 event.

**Conclusion:**

Perioperative desensitization was associated with fewer AMR and deaths after HTx, and efficacy was more pronounced in men than women.

## Introduction

Heart transplantation (HTx) is the only curative treatment for advanced heart failure (1). Immunological sensitization affects cardiac allograft longevity; and recipients at high immunological risk present an increased risk of early postoperative cardiac graft dysfunction, antibody-mediated rejection (AMR), and death (2).

Sensitization refers to a state, in which HLA antibodies are circulating in the blood of recipients prior to transplantation. The means of sensitization include pregnancy, blood transfusions, previous heart transplantations, and mechanical assistance devices such as left ventricular assistance devices (3).

The presence of preformed donor-specific antibodies (pfDSA) in a recipient patient characterizes sensitization and needs to be accounted for when considering perioperative immunological induction strategies, as well as postoperative desensitization (3–8). Indeed, exclusive postoperative desensitization procedure after HTx seemed to benefit sensitized patients, as they presented similar overall survival as compared to contemporary patients without pfDSA (9).

Several elements point towards a higher immunological risk of pregnancy-induced sensitization as compared to other causes of sensitization (4). The present work aimed to assess whether the cause of sensitization affected the efficacy of perioperative desensitization procedures. In a historical cohort comparison analysis in sensitized patients, we compared those who benefited from desensitization (after it was implemented), to those with similar immunological risk who did not (before the protocol was implemented). Subgroup analyses then assessed between-group differences based on gender and pregnancy.

## Methods

This is a retrospective analysis comparing two cohorts of HTx recipients. We included all consecutive sensitized HTx recipients, three years around the date of implementation of desensitization procedures (01/2007), in a high-volume heart transplantation center. Patients were considered sensitized when presenting with pfDSA on the day of HTx, with mean fluorescence intensity above 1000 units. We excluded patients with combined transplantation procedures (i.e. kidney and heart, or liver and heart).

Our institutional review board approved the protocol, informed consent was obtained at listing, and data were collected as part of the HEARTS registry (clinicaltrials.org identifier NCT03393793). The study was in strict compliance with the International Society for Heart and Lung Transplantation (ISHLT) ethics statement.

### Study Outcomes and Definitions

The main study endpoint was a composite of death and biopsy-proven antibody-mediated rejection (AMR) up to 5-years follow-up. Furthermore, subgroup analyses were performed based on gender and cause of sensitization. Secondary analyses included the analysis of the composite endpoint at 1-year follow-up, and analyses on death and AMR separately at 5-years follow-up. Sensitivity analyses included postoperative 30-days mortality as endpoint.

Following current recommendations, diagnosis of graft rejection required histological confirmation on endomyocardial biopsy (EMB) specimens. Routine EMB protocol remained similar during all the study period and consisted of 3 biopsies per month starting on day 15 until day 65 after HTx, then once every 20 days until four months, then monthly until six months, then once every 45 days until year 1. After year 1, they were performed every four months until year 3 and then every eight months until year 5. An additional EMB was performed when in presence of indirect signs of rejection (decrease in left ventricular ejection fraction, acute arrhythmia, or clinical indication). All biopsy specimens were processed and examined according to current standards, requiring retrospective analyses, from frozen samples in some cases (10, 11). Standard serial sections were cut from formalin-fixed paraffin-embedded EMB specimens and stained with hematoxylin-eosin-saffron for diagnosis. Immunofluorescence for C4d was performed on all specimens (frozen section; C4d monoclonal 1/100; Quidel Corporation; Polyclonal rabbit anti-mouse fluorescein isothiocyanate; Dako). Only capillary staining for C4d was assessed.

### Standard Immunosuppressive Protocol

Except for postoperative desensitization, the standard immunosuppressive regimen did not change during the study period (2004-2010). As previously described (12), in the postoperative period it included thymoglobulin induction therapy from day 0 to day 4 (rabbit ATG, Thymoglobuline, Genzyme, Lyon, France), methylprednisolone bolus infusion on day 1, ciclosporine after day 1, and mycophenolate mofetil (MMF) after day 4. Corticosteroids were converted to oral form starting on day 4, with a dosage of 1 mg/kg initially. They were progressively lowered to reach 20 mg/day at 2 months and 5 mg/day after the anniversary date of HTx. Ciclosporine was the only calcineurin-inhibitor used at the time, and trough targets depended on delay since transplantation.

### Intervention: Perioperative Desensitization Procedure Around HTx

Starting in 2007, a dedicated protocol was performed in sensitized patients, i.e. transplanted with pfDSA with MFI above 1000. It included plasmaphereses, with 1 session immediately before HTx and 4 after, for a total of 5 sessions (with a 1:1 fresh frozen plasma/albumin ratio), then IVIg (2 g/kg over a four-day period starting on day 5).

### Anti-HLA Antibodies Detection

The detection of anti-HLA antibodies was based on Luminex Single Antigen beads technology (One Lambda, Canoga Park, CA). The fluorescence of each bead is detected by a reader and recorded as the normalized mean fluorescence intensity (MFI) (13). Retrospective cross matches were performed by complement-dependent cytotoxicity (CDC) assay on T- B-donor lymphocytes with historical and current sera. HLA typing of heart recipients was performed by low-resolution class I HLA-A, -B, and class II HLA-DR, -DQ PCR-SSO (LABType, One Lambda). Donor HLA-A, B, DR, DQ typing was performed by CDC (One lambda tissue-typing trays) and controlled by molecular biology. Because this technology was routinely deployed after the date of HTx of some patients, it was performed retrospectively on samples that had been stored to that effect, guaranteeing the homogeneity of pfDSA assessment.

In the present study, pFDSA were considered positive (i.e. recipient patient was sensitized), only if they presented a mean fluorescence intensity (MFI) above 1000. Cumulative DSA (cDSA) was computed as the sum of MFI of all DSA. Only patients with positive pfDSA on the day of HTx, confirmed with retrospective analyses, were included in the present study.

### Standard AMR Treatment

AMR was treated with high-dose intravenous corticosteroids (1 g/day for 3 days) concomitantly to plasmaphereses sessions over 5 days then intravenous immunoglobulins (IVIg) (2 g/kg over a 4-day period) were administered. Thereafter, oral corticosteroids were then increased to 1 mg/kg for 10 days, with progressive weaning afterward.

### Statistical Analyses

Continuous variables are described as median (interquartile range) and categorical variables as number (percentage). Fischer’s exact tests were performed to compare proportions between groups. Cumulative survival curves for the time-to-event analyses were constructed according to the Kaplan-Meier method. Cox regression was used to assess the association between treatment and clinical outcomes. The alpha risk was set to 5%. All calculations were performed using SPSS v22.0 (IBM, Armonk, USA).

## Results

**Figure 1:**
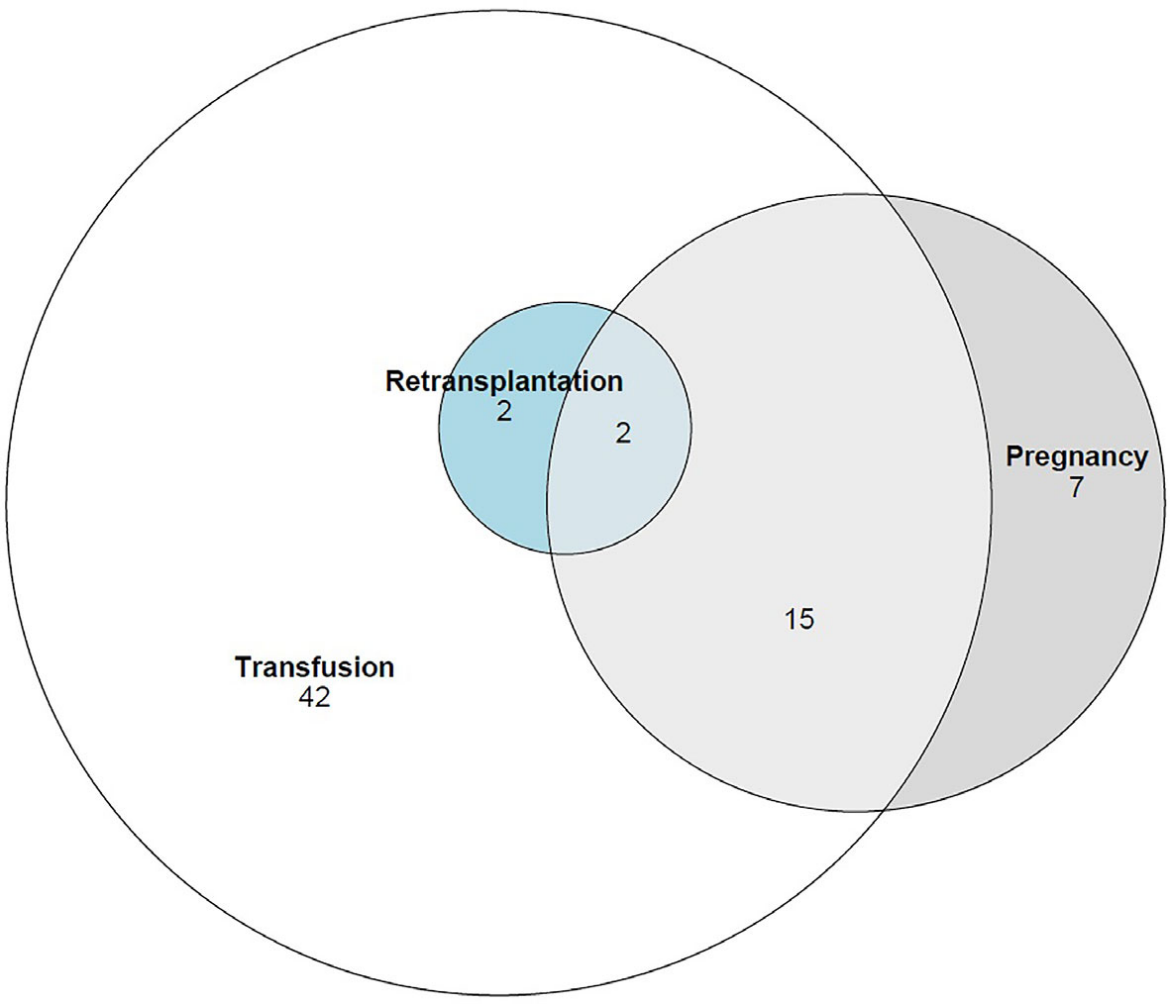
Causes of sensitization in the overall cohort.

Overall, 68 sensitized patients were included with 37 patients in the desensitized group and 31 patients in the historic control group. They were 48.4 (interquartile range: 36.2;54.9) years-old, with 33/68, 48.5% women and 12/68, 17.6% under preoperative extracorporeal life support (ECLS). The cause of sensitization was pregnancy in 24/68, 35.3%, transfusion in 61/68, 89.7%, and previous HTx in 4/68, 5.9% (overlaps are presented in **Figure 1**). Baseline characteristics are presented in **Table 1**.

**Table 1:** Baseline characteristics.

3	Overall (n=68)	Control (n=31)	Treated (n=37)	*p-value*
**Women**	33 (48.5%)	13 (41.9%)	20 (54.1%)	0.34
**Age, median, [IQR], in years**	48.4 [36.2;54.9]	50.4 [38.5;55.6]	45 [35.4;54.4]	0.45
**Height, median, [IQR], in cm**	167.5 [162;175]	171 [162;175]	167 [163;172]	0.41
**Weight, median, [IQR], in kg**	65 [58.5;78.5]	68 [59;82]	63 [56;78]	0.17
**Cause of heart failure**				0.54
**Dilated cardiomyopathy**	34 (50.0%)	17 (54.8%)	17 (45.9%)	
**Ischemic cardiopathy**	19 (27.9%)	9 (29.0%)	10 (27.0%)	
**Other**	15 (22.1%)	5 (16.1%)	10 (27.0%)	
**LVAD or TAH**	12 (17.6%)	5 (16.1%)	7 (18.9%)	1.0
**Preoperative ECMO**	12 (17.9%)	3 (9.7%)	9 (24.3%)	0.11
**Cause of sensitization**				0.45
**pregnancy**	24 (35.3%)	8 (25.8%)	16 (43.2%)	
**transfusion subgroup**	61 (89.7%)	28 (90.3%)	33 (89.2%)	
**previous HTx**	4 (5.9%)	1 (3.2%)	3 (8.1%)	
**Cumulative DSA MFI, [IQR], in u**	4031 [2074;12083]	4026 [1788;8725]	4560 [3162;13392]	0.28
** pregnancy-related subgroup**	11231 [3894;20727]	13372 [4643;33728]	10233 [3896;16063]	0.42
** transfusion subgroup**	4026 [1943;9035]	3515 [1787;8651]	4034 [2724;11183]	0.35
** previous HTx subgroup**	15960 [12023;19054]	8576 [na]	16450 [15470;21657]	0.50

ECMO, extracorporeal membranous oxygenation; HTx, heart transplantation; IQR, interquartile range; LVAD, left ventricular assistance device; TAH, total artificial heart.

There were no significant differences in baseline characteristics between the two groups. In particular, patients in the treated group were under similar ECLS support, as compared to those in the historic control group (9/37, 24.3% *vs.* 3/31, 9.7%, *p*=0.20). There was no significant difference in women proportion (20/37, 54.1% *vs.* 13/31, 41.9%, *p*=0.34), nor pregnancy-induced sensitization (16/37, 43.2% *vs* 8/31, 25.8%, *p*=0.20). In desensitized women, pregnancy-induced sensitization proportion was similar as in women in the historic control group (16/20, 80% *vs* 8/13, 61%, *p*=0.42). Causes of sensitization were similar in the two groups (*p*=0.45). Cumulative pfDSA were similar between the two groups [4026 (1788;8725) *vs* 4560 (3162;13392) MFI units, *p*=0.28]. DSA included class II anti-HLA antibodies in the same proportion in the two groups (19/31, 61.3% *vs* 26/37, 70.3%, *p*=0.45).

Cumulative preformed DSA were higher in women than in men [8576 (3972;16450) *vs* 3004 (1600;4984) MFI units, *p*<0.0001]. In women (n=33), cumulative preformed DSA were higher in those sensitized because of pregnancy than other women [4978 (4027;11183) *vs* 11231 (3896;20727), p<0.0001].

### Effect of Desensitization on Clinical Outcomes, in the Overall Cohort

**Figure 2:**
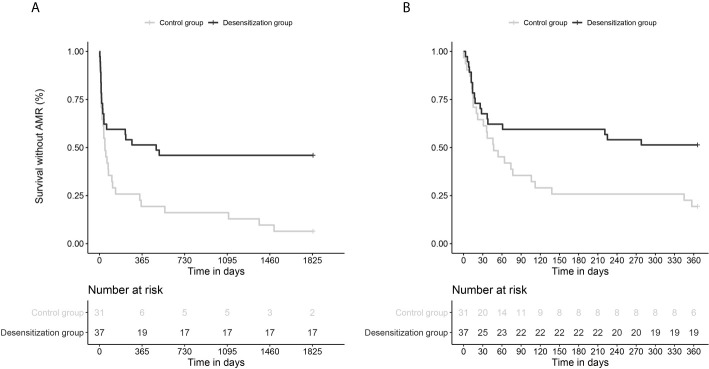
Survival curves comparing the incidence of the main composite endpoint (death and AMR) in desensitized patients and historic control patients, with a 5-year follow-up **(A)** and 1-year follow-up **(B)**. **(A)**. Multivariable Cox survival analyses (with cumulative DSA, age, desensitization intervention, and sex) confirmed desensitization was independently associated with fewer events with an adjusted (adj.) HR = 0.44 (0.25-0.79), p = 0.006; the other independent variable being cumulative DSA [per 1000 MFI-increase, adj. HR = 1.031 (1.005-1.057), p = 0.018]. **(B)**. Multivariable Cox survival analyses (with cumulative DSA, desensitization intervention) yielded a significant association between desensitization and the primary outcome with 1-year follow-up, adj.HR = 0.49 (0.27-0.91), p = 0.023) and cumulative DSA also independently associated with the primary outcome [per 1000-MFI increase, adj.HR=1.028 (1.002-1.053), p = 0.031].

Desensitization was associated with a significant reduction in the incidence of the main composite outcome (death or AMR) with 29/31, 93.5% *vs*. 20/37, 54.1% of patients during the five-year follow-up; corresponding in survival analyses to an unadjusted (unadj.) hazard-ratio (HR) of 0.42 (0.23-0.74), *p*=0.003 (see **Figure 2A**). Multivariable Cox survival analyses (accounting for cumulative DSA, age, desensitization intervention, and sex) confirmed desensitization was independently associated with fewer events with an adjusted (adj.) HR=0.44 (0.25-0.79), *p*=0.006; the other independent variable being cumulative DSA (per 1000 MFI-increase, adj. HR=1.031 (1.005-1.057), *p*=0.018).

**Figure 3:**
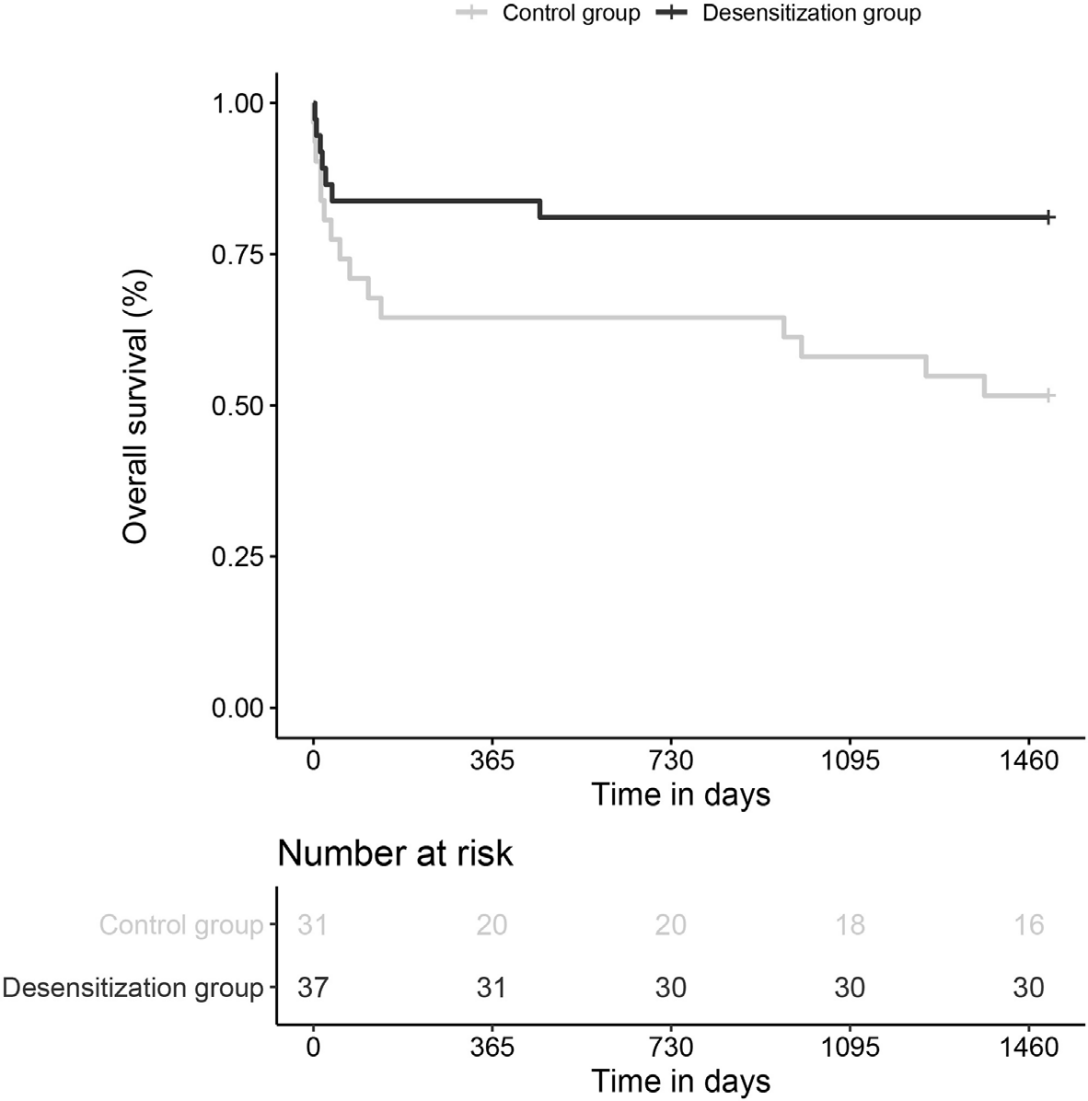
Survival curves comparing the incidence of death in desensitized patients and historic control patients, with a 5-year follow-up.

Secondary analyses showed the benefit of desensitization against death, with a significant protective association (7/37, 18.9% *vs* 15/31, 48.4%, unadj.HR=0.34 (0.14-0.85), *p*=0.02) (see **Figure 3**), and a trend against AMR (16/37, 43.2% *vs* 17/31, 54.8%; unadj.HR=0.51 (0.26-1.03), *p*=0.06). Multivariable analyses accounting for cumulative DSA yielded similar results.

### Effect of Desensitization: Comparing Men and Women

**Figure 4:**
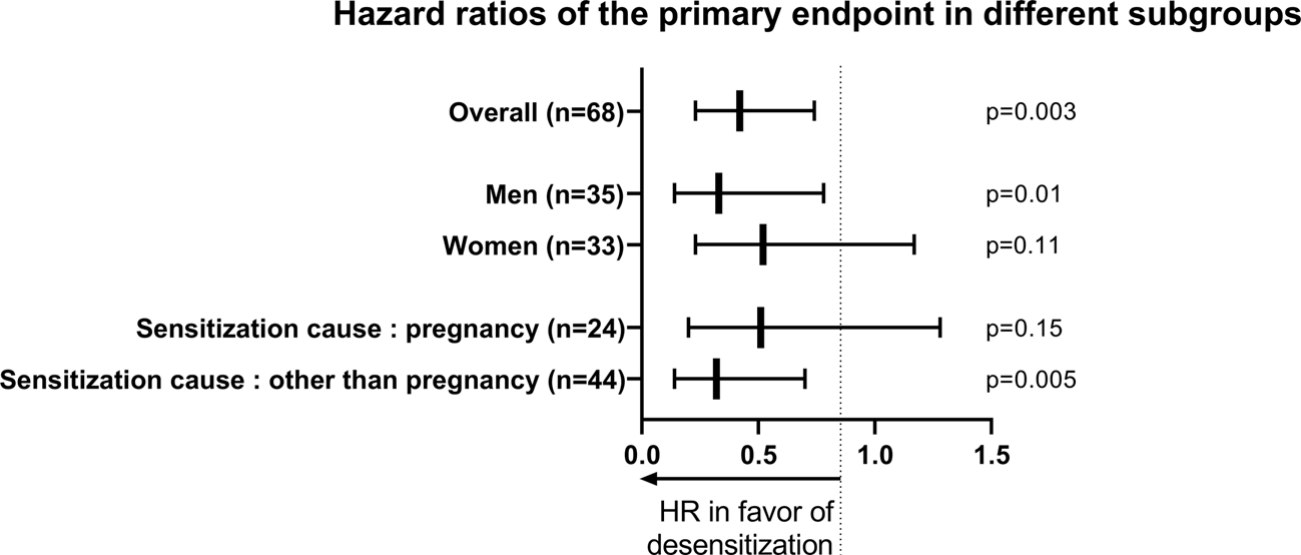
Forest plot comparing the efficacy of desensitization in different subgroups, on the incidence of the main composite endpoint (death and AMR).

Subgroup analyses were performed (see **Figure 4**). In men, desensitization was associated with fewer events [8/17, 47.1% *vs*. 17/18, 94.4%; unadj.HR=0.33 (0.14-0.78); p=0.01]. In women, desensitization was not significantly associated with fewer events [12/20, 60% *vs*. 12/13, 92.3%; unadj.HR=0.52 (0.23-1.17), p=0.11]. In terms of the number of subjects to treat, in men, 2.1 patients treated prevented one event, while in women, 3.1 need to be treated.

### Effect of Desensitization: Comparing Pregnancy-Induced to Other Causes of Sensitization

In women patients who are sensitized because of pregnancy (n=24), desensitization was not significantly associated with fewer events [11/16, 68.8% *vs*. 8/8, 100%, unadj.HR=0.51 (0.20-1.28), p=0.15].

In other causes of sensitization (n=44), desensitization procedure was associated with fewer events [unadj.HR=0.32 (0.14-0.70), p=0.005]. In details, in patients with a history of transfusion (n=61), unadj.HR was 0.40 (0.22-0.74), p=0.003), in patients with previous HTx (n=4), there was no association with the primary endpoint (p=0.62) (see **Figure 3**).

### Additional Sensitivity Analyses

The association between desensitization intervention and the incidence of the primary composite endpoint (death and AMR) at one-year follow-up was assessed. These analyses confirmed that there was a significant reduction of deaths and AMR in patients who underwent desensitization as compared to those who did not [unadj.HR=0.49 (0.26-0.89), p=0.02]. Multivariable Cox survival analysis also confirmed the independence of association between desensitization and event reduction [adj.HR=0.49 (0.27-0.91), p=0.023]. The other independent variable associated with the events was cumulative DSA [per 1000-MFI increase, adj.HR=1.028 (1.002-1.053), p=0.031] (see **Figure 2B**).

Focusing on postoperative mortality (assessed by 30-days mortality), there was no significant difference between the two groups (5/37, 13.5% in the treated group *vs* 6/31, 19.4% in the control group, p=0.33). Causes of postoperative deaths were rejection in 2/5, 40.0% in treated patients *versus* 4/6, 66.7% in control patients; and sepsis in 2/5, 40.0% in treated patients *versus* 1/6, 16.7% in control patients (p-value could not be computed due to the limited number of events). Finally, there was one case of fatal hemorrhage in the treated group (*versus* none), and one case of fatal stroke in the control group (*versus* none).

### Exploratory Analyses

Association between the presence of class II anti-HLA antibodies and the primary outcome, with a 5-year follow-up, was assessed and did not yield significant association. Interaction analyses between preformed cumulative DSA and desensitization procedures, regarding the primary outcome, with a 5-year follow-up, also did not yield significance, although it may be due to lack of power.

## Discussion

Our study yielded three main findings: i) desensitization procedures were associated with fewer deaths and AMR; ii) the benefit of desensitization was not equal between men and women; and, iii) preformed cumulative DSA was independently associated with deaths and AMR, after adjusting on desensitization procedures.

Sensitization is a major challenge because it restricts access to organ transplantation, due to the limited available donor pool and increasing wait time. Post-transplantation outcomes are less favorable in sensitized than in non-sensitized recipient patients, which may topple the risk-benefit balance towards treatment abstention in some of these patients at high immunological risk (3, 10).

Sensitized patients (i.e. patients with pfDSA) present more adverse outcomes after HTx, with more deaths and AMR than patients without pfDSA (7). The presence of pfDSA is mostly dependent on a previous sensitization event, for which main causes are blood transfusion, pregnancy, and previous transplantation (14). Furthermore, we previously reported that pregnancy-induced pfDSA was associated with more AMR than those related to other means of sensitization (4). In a recent statement, the American Heart Association emphasized the need to better characterize patients who would best benefit from desensitization procedures, which we humbly tried to partially address in the present paper (3). Our team previously described the benefit of performing postoperative desensitization (9).

In the present paper, a historical comparison was performed, examining the time of implementation of desensitization procedures in sensitized patients. The results confirmed the efficacy of these procedures, with a reduced incidence in AMR and deaths after HTx in the overall cohort. However, subgroup analyses showed that sensitization due to previous pregnancy was less beneficial for desensitization procedures than sensitization due to other causes (previous heart transplantation and blood transfusions). Indeed, the impact of desensitization procedures appeared to be less significant in this subgroup. Moreover, the efficacy of desensitization was less significant in women as compared to men (in terms of the number of subjects to treat to prevent one event, 3.1 women *vs*. 2.1 men) (15). The reasons for these findings may include the fact that women present more pfDSA than men (14) and that for a given quantity of pfDSA, they are more at risk of developing AMR afterward (4). Gender difference in solid organ transplant recipients regarding de-novo HLA antibodies production is unclear. In a previously described cohort of 463 patients, we did not observe any difference between women and men regarding *de novo* DSA production (4). Yet, in another study, in 47 patients after vaccination, women seemed more exposed to the increase of non-specific HLA antibodies than men (16). Regardless, the hypothetical means to address this issue of gender-difference in desensitization efficacy may rely on intensification of desensitization procedures in sensitized women with previous pregnancies or escalating maintenance immunosuppression (17, 18). In our study, the lack of significant difference of postoperative mortality (13.5% in the treated group *vs* 19.4% in the control group) may be due to the limited sample size.

A strength of this study is that all *sera* were analyzed to assess DSA, even for the study period in which Luminex based analyses were not standard of care, for which, samples had been stored to that effect. Similarly, endomyocardial biopsies were all retrospectively re-analyzed to uphold the most recent standards. We also acknowledge several limitations to the present work. The retrospective monocentric design of the study, with historical comparison, means that future studies will need to externally validate the results found. Likewise, the relatively small number of patients demands confirmation. Indeed, proper interaction analyses to assess between-group-effect on treatment-effect could not be performed on relatively few patients (however difficult that may be in the field of heart transplantation). Furthermore, patients could present multiple causes of desensitization, however, the small number of patients could not allow subgroup analyses between overlapping causes, which may be more feasible in larger transplanted cohorts such as those in renal transplantation (14). We could not assess the efficacy of desensitization on DSA after HTx, specifically *de novo* DSA, due to the lack of *sera* in the historical control cohort. Maintenance immunosuppression regimen has been subject to change in the past 15 years with tacrolimus suggested instead of ciclosporin (19) and mammalian target of rapamycin (mTOR) inhibitors in addition to, or in place of, calcineurin inhibitors (12, 20). These may impact the treatment effect observed due to desensitization. However, maintenance immunosuppression is more associated with later outcomes than those occurring before the first year postoperative, whereas desensitization may be associated with a treatment effect that is more important for first-year outcomes than five-year outcomes (as attested by sensitivity analyses). While the difference was not significant, the historic control cohort did not present the same preoperative risk, being less severe compared to the treated cohort; however, the results point towards better outcomes in the treated cohort, comforting the efficacy of desensitization despite worse preoperative odds. Finally, this latter point also emphasizes that patients who would not otherwise have undergone HTx due to high operative and immunological risk did benefit from transplantation thanks to specific desensitization procedure, and did so with acceptable risk in this retrospective analysis. These findings may help alleviate immunological risk in sensitized patients, with dedicated perioperative desensitization procedures.

## Conclusion

In this retrospective study of sensitized patients at the time of HTx, desensitization protocol was associated with a significant reduction in death and AMR. The cause of sensitization affected the efficacy of the protocol, with a less significant effect in women. These results warrant further research in patients at higher-immunological risk, to promote gender equality after HTx.

## Data Availability Statement

The raw data supporting the conclusions of this article will be made available by the authors, without undue reservation.

## Ethics Statement

The studies involving human participants were reviewed and approved by Pitié-Salpétrière Comité de Protection des Personnes. The patients/participants provided their written informed consent to participate in this study.

## Author Contributions

LN wrote the manuscript and performed all analyses. J-ES, M-CB, GC, JA, AB, CS, V-DK, MC, PR, MK, SV, PL, and SS participated to data collection and patient follow-up, and provided critical review to the manuscript. All authors contributed to the article and approved the submitted version.

## Funding

Funding was granted by LFB laboratories (Les Ulis, France).

## Conflict of Interest

The authors declare that the research was conducted in the absence of any commercial or financial relationships that could be construed as a potential conflict of interest.
