# Supplementary material for: Impact of Sex in the Efficacy of Perioperative Desensitization Procedures in Heart Transplantation: A Retrospective Cohort Study
Source: Front Immunol. 2021 Jul 7;12:659303. doi: 10.3389/fimmu.2021.659303 (PMC8292826; doi:10.3389/fimmu.2021.659303)

Additional Table. Cumulative preformed donor-specific anti-HLA antibodies on day 0 (in mean immunofluorescence units)

Control	Desensitized
4984	1822
2955	9035
6669	1768
1089	3162
1005	3463
1153	4978
1788	38922
41620	4809
2616	15676
1785	4866
17984	3820
1600	11183
4027	7483
23263	32530
9480	21657
8725	4113
2589	1550
2180	23469
83836	1308
4027	3614
4026	1534
6525	2724
1164	15470
25835	6010
3004	3483
1397	12982
1943	4560
13938	16450
1865	16429
6054	1967
8576	1180
	13392
	4034
	17145
	3882
	2276
	3972

Additional Figure. Bar chart representing the proportion of patients who developed the composite outcome during the 5-years follow-up, depending on the number cumulative preformed donor-specific anti-HLA antibodies mean immunofluorescence units.

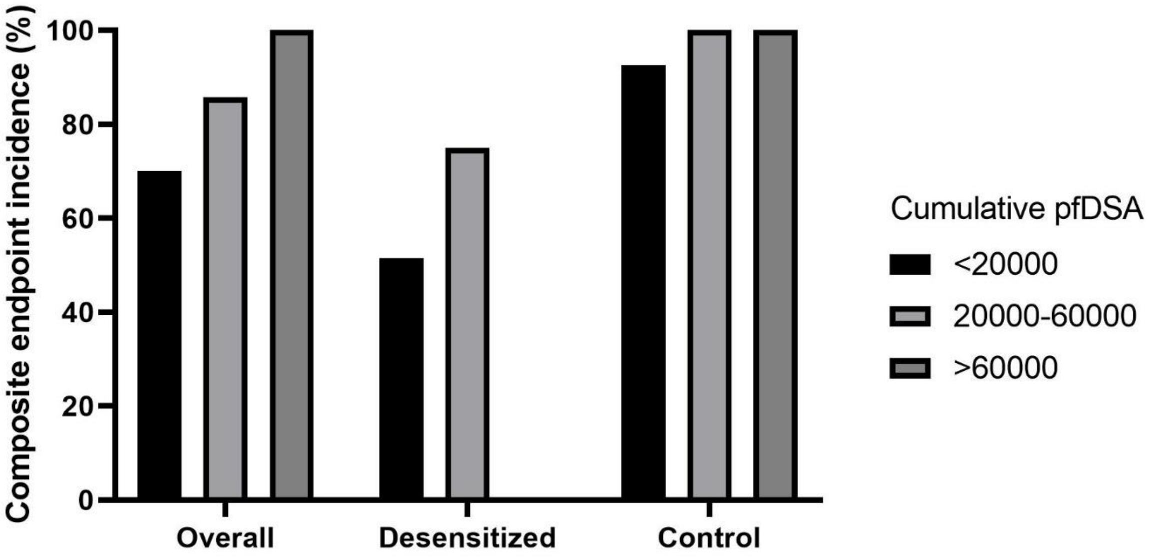

Supplement: Supplementary file 1 [file DataSheet_1.pdf]
